# Unveiling *Curvularia tuberculata*-induced leaf anomalies in *Rhododendron ferrugineum*: implications in cultural-ecological conservation and harnessing microbial intervention in socio-economic advancement

**DOI:** 10.3389/fmicb.2023.1280120

**Published:** 2024-01-11

**Authors:** Juhita Dhar, Aishee Hazra, Riddhisha Patra, Varun Kumar, Vetriselvan Subramaniyan, Vinoth Kumarasamy, Arup Kumar Mitra, Amany A. Sayed, Lotfi Aleya, Fatma M. El-Demerdash, Mikhlid H. Almutairi, Shopnil Akash, Mohamed M. Abdel-Daim, Achal Kant, Bikram Dhara

**Affiliations:** ^1^Department of Microbiology, St. Xavier’s College (Autonomous), Kolkata, West Bengal, India; ^2^Birla Institute of Technology and Science, Pilani, Rajasthan, India; ^3^Pharmacology Unit, Jeffrey Cheah School of Medicine and Health Sciences, Monash University, Bandar Sunway, Selangor, Malaysia; ^4^Center for Transdisciplinary Research, Department of Pharmacology, Saveetha Institute of Medical and Technical Sciences, Saveetha Dental College and Hospital, Saveetha University, Chennai, India; ^5^Department of Parasitology and Medical Entomology, Faculty of Medicine, Universiti Kebangsaan Malaysia, Kuala Lumpur, Malaysia; ^6^Zoology Department, Faculty of Science, Cairo University, Giza, Egypt; ^7^Chrono-Environnement Laboratory, UMR CNRS, Franche-Comté University, Besançon, France; ^8^Department of Environmental Studies, Institute of Graduate Studies and Research, Alexandria University, Alexandria, Egypt; ^9^Zoology Department, College of Science, King Saud University, Riyadh, Saudi Arabia; ^10^Department of Pharmacy, Daffodil International University, Dhaka, Bangladesh; ^11^Pharmacology Department, Faculty of Veterinary Medicine, Suez Canal University, Ismailia, Egypt; ^12^Narayan Institute of Agricultural Sciences, Gopal Narayan Singh University, Sasaram, India; ^13^Center for Global Health Research, Saveetha Medical College and Hospital, Saveetha Institute of Medical and Technical Sciences, Chennai, India

**Keywords:** plant pathology, biotherapeutics, microbe-assisted bioremediation, synthetic biology, leaf infection, *R. ferrugineum*, *C. tuberculata*, cold-adapted pathogen

## Abstract

**Introduction:**

The research focuses on *Rhododendron ferrugineum* L., Nepal’s national flower and Uttarakhand’s state tree, thriving in high-altitude mountain ecosystems.

**Methodology and Result:**

A study conducted in Himachal Pradesh (Latitude: N 31° 6’ 2.0088”, Longitude: E 77° 10’ 29.9136”) identified leaf anomalies resembling rust-like manifestations in *R. ferrugineum*. These anomalies were traced back to the pathogenic fungus *Curvularia tuberculata*, marking the first documented case of its impact on *R. ferrugineum* in India.

**Discussion:**

This discovery emphasizes the need for vigilant monitoring, disease management research, and conservation efforts to protect the cultural and ecological significance of this iconic shrub. Beyond its immediate findings, the study introduces a novel dimension to Indian flora by associating *C. tuberculata* with *R. ferrugineum*, historically linked to monocotyledonous crops. The research methodology combines traditional microscopic examination with advanced genomic sequencing and phylogenetic analysis, enhancing pathogen identification accuracy.

**Future prospect:**

In a broader context, this research aligns with the United Nations Sustainable Development Goals (SDGs) by highlighting the importance of environmental preservation, conservation, and sustainable management. It underscores the intricate interplay between biodiversity, cultural heritage, and the need for holistic solutions. Overall, this study calls for proactive measures to protect *R. ferrugineum’s* cultural and ecological heritage and emphasizes the significance of interdisciplinary approaches in addressing emerging ecological threats.

## 1 Introduction

Global ecosystems, encompassing diverse landscapes from agricultural fields to natural floral ecosystems, are confronted with an ever-evolving and intricate challenge posed by pathogenic fungi. This challenge is particularly pronounced in India, a country renowned for its ecological diversity characterized by a wide range of climatic zones and an abundance of native flora. Within this context, India has become a pivotal focal point for the observation and study of these complex ecological interactions. One of the pivotal factors amplifying the impact of pathogenic fungi on India’s ecosystems is the changing environmental conditions. Specifically, the escalation of temperatures and the intensification of heatwaves have significantly heightened the vulnerability of India’s native flora. Regions such as the Himalayan foothills, with their unique ecosystems, and the biodiverse expanse of the Western Ghats, are experiencing a surge in disturbances that not only disrupt the delicate ecological balance but also wield substantial economic consequences for regional agriculture and associated industries (Chaloner et al., 2021). An alarming and noteworthy facet of this narrative is the emergence and adaptability of trans-kingdom fungi, a phenomenon that transcends conventional taxonomic boundaries. These pathogens, primarily originating from the *Ascomycota* and *Glomeromycota* phyla (Gauthier and Keller, 2013), possess an extraordinary capability to infect hosts spanning multiple kingdoms. This remarkable adaptability creates a scenario where plant species once considered resistant are now under the looming threat of fungal infections. Moreover, some of these fungi have displayed the disconcerting tendency to affect human hosts, thereby introducing a new dimension to this ecological challenge and raising significant concerns for public health. To delve even further into the complexity of this issue, it is crucial to recognize the multifaceted nature of the interactions between pathogenic fungi and their hosts. The mechanisms that underlie fungal adaptability and host susceptibility are intricate and multifarious, encompassing genetic, physiological, and environmental factors. Consequently, the urgent imperative lies in conducting comprehensive research, proactive conservation efforts, and innovative strategies to mitigate the multifaceted impact of pathogenic fungi on ecosystems, agriculture, and human well-being. In conclusion, the challenges posed by pathogenic fungi in global ecosystems, with a specific emphasis on India’s unique ecological diversity, demand in-depth exploration and comprehensive understanding. This includes elucidating the mechanisms of fungal adaptability, identifying host susceptibility factors, and devising innovative solutions to safeguard not only India’s rich biodiversity but also its agricultural sustainability and public health.

**Figure 1:**
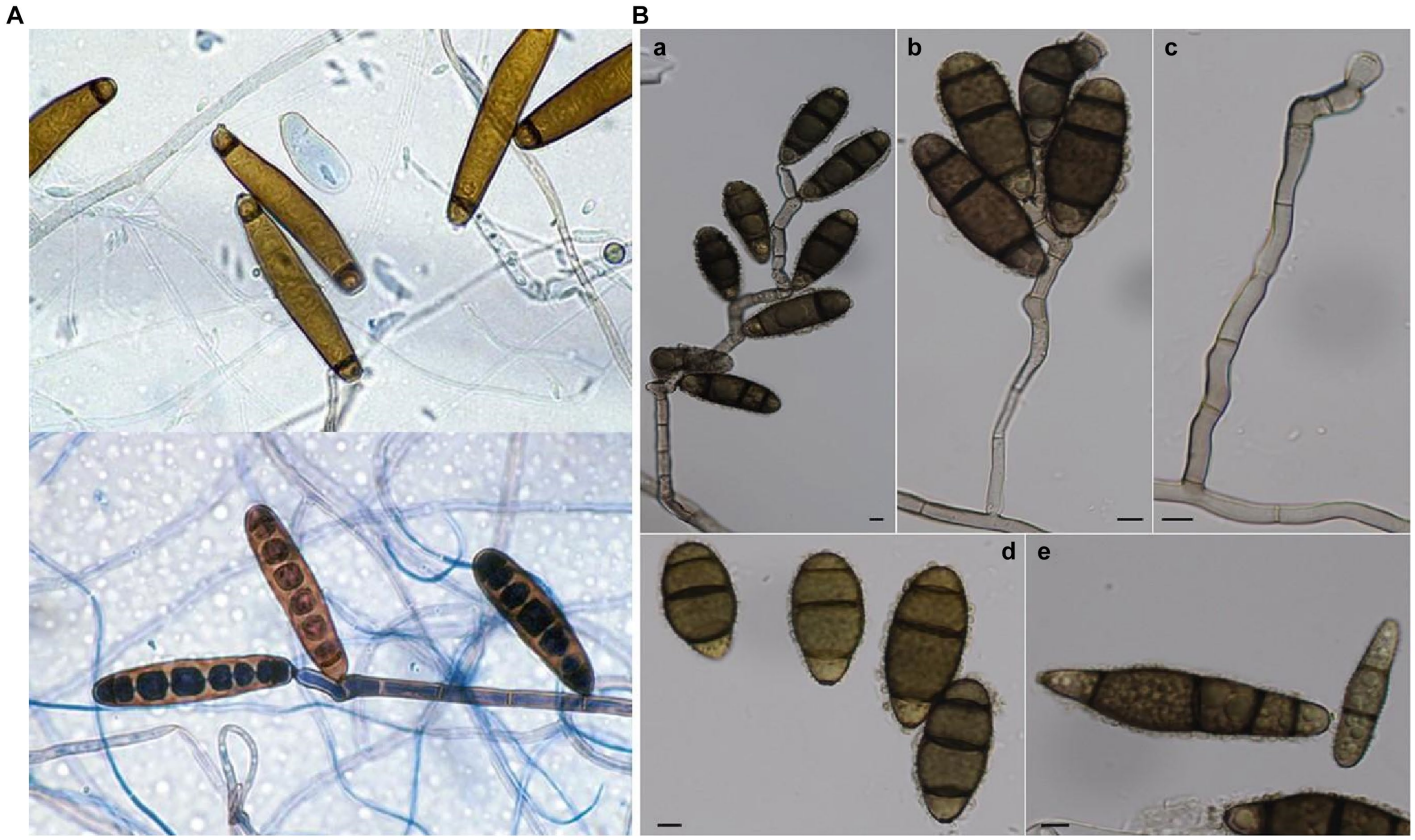
**(A)** Conidia of *E. rostratum* & Sympodially elongating conidiophore and conidia of *E. rostratum* [taken from open access source, ref.: (Domsch et al., 1980; Haq et al., 2020)]; **(B)** *C. tuberculata*: **(A–C)** Conidiophores and conidia. **(D,E)** Conidia (Bars = 5 μm, unless otherwise specified). [taken from open access source, ref.: (Jain, 1962; Zhang and Watson, 1997)].

The mechanics underlying these fungal incursions are both fascinating and concerning. From an ecological standpoint, the dispersal methods of these fungi—via wind, water, or animal vectors—are critical (Epstein and Nicholson, 2006). The initial adherence of spores to potential host surfaces, driven by specialized adhesive molecules, often heralds the onset of an infection cycle. This subsequently evolves into a complex interplay of germination, penetration, and establishment within the host. Two fungal species stand out in this emerging narrative: *Exserohilum rostratum* and *C. tuberculata* (Figure 1). While their natural behavior as soil saprophytes (Doehlemann et al., 2017) has been documented, it’s their newly observed pathogenic tendencies, especially towards an array of Indian plant species, that demand attention. Plants located close to human settlements are becoming inadvertent vectors for these crossover pathogens, complicating both ecological balance and public health considerations (Rinaldi et al., 1987). Our research has made a significant and pioneering contribution to the field of ecology and plant-pathogen dynamics, with a recent breakthrough discovery in the state of Himachal Pradesh, India. In this region, we have identified a novel host-pathogen interaction that has far-reaching implications. Specifically, the pathogen *C. tuberculata*, which has traditionally been associated with staple crops, is now encroaching upon the habitat of the iconic *R. ferrugineum*. However, its importance extends beyond ecological considerations; it serves as a symbol of cultural pride and represents a substantial portion of global biodiversity. Under the Rhododendron genus, there are a staggering 800 to 1,100 species (Bursill and Rouse, 1998), showcasing the diversity and richness of this plant family. Furthermore, the significance of *R. ferrugineum* transcends national borders, as it is not only recognized as the national flower of Nepal but also stands as an emblem of Himachal Pradesh’s natural heritage (Kumar et al., 2019; Namgay and Sridith, 2020).

The colloquial name for *R. ferrugineum*, the ‘Alpine rose,’ evokes the image of a hardy and resilient plant that has thrived in challenging mountainous environments. Beyond its ecological and cultural importance, this species has a rich history intertwined with human traditions and traditional medicine practices (Popescu and Kopp, 2013). Historically, various parts of *R. ferrugineum* have been employed in the treatment of a wide array of ailments, ranging from arthritis to rheumatism (Hubert et al., 2022). However, the utilization of this plant for medicinal purposes also underscores the need for a careful and balanced approach. Its potential for adverse reactions (Louis et al., 2010) necessitates a nuanced understanding of its properties and the responsible use of its medicinal attributes.

Delving into the phytopathogenic tactics, these fungi exhibit a broad arsenal from enzyme-driven cellular breaches to specialized toxin production, all strategically orchestrated to compromise host defenses (Meng et al., 2009). *Curvularia* sp., as filamentous fungi with wide distribution and ecological significance, were the focus of our evolutionary analysis. We delved into the ITS sequences of various *Curvularia* isolates to unravel their evolutionary relationships. Additionally, we integrated bootstrap values to assess the reliability of the inferred phylogenetic relationships. The urgency of our study is further underscored by the previously documented predilection of *C. tuberculata* for North Indian crops (Majeed et al., 2016). In conclusion, our research has unveiled a fascinating and intricate host-pathogen interaction in Himachal Pradesh, shedding light on the evolving dynamics between *C. tuberculata* and *R. ferrugineum*. Beyond the ecological implications, this discovery highlights the cultural, biodiversity, and traditional medicinal significance of *R. ferrugineum*, emphasizing the need for both conservation efforts and responsible utilization of this invaluable resource. This newfound knowledge opens avenues for further research and underscores the interconnectedness of ecological, cultural, and medicinal aspects of plant species like *R. ferrugineum* in our natural world. By synthesizing historical data with contemporary findings, this research seeks to provide a holistic perspective on a novel fungal invasion, offering insights into its mechanisms, ecological ramifications, and potential countermeasures.

## 2 Materials and methods

### 2.1 Isolation and cultivation of pathogens from *Rhododendron ferrugineum* leaves

#### 2.1.1 Sample procurement and preservation

**Figure 2:**
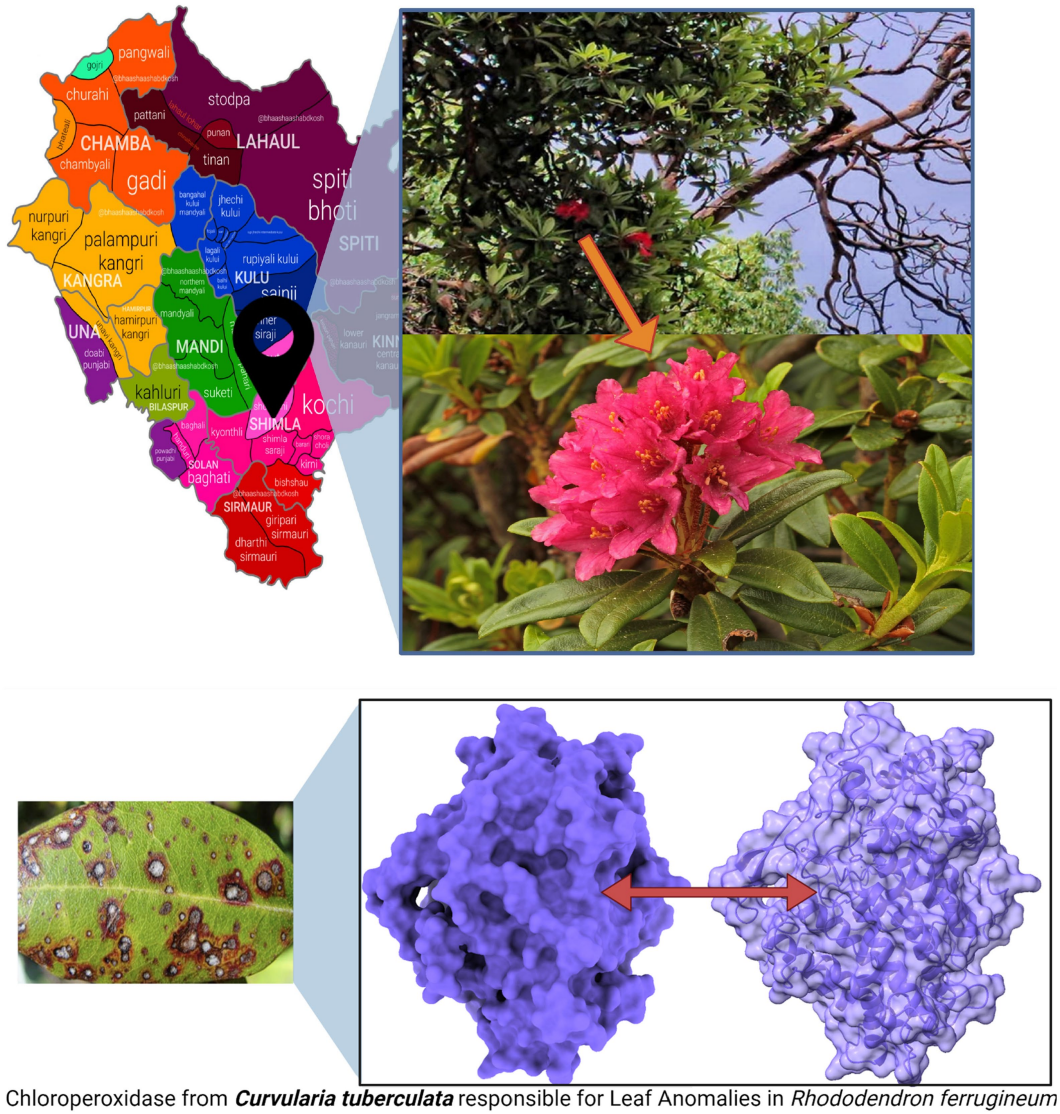
*R. ferrugineum* plants from where the infected leaves were collected, at summer hill, Shimla, Himachal Pradesh, India, and pathogenic protein of *C. tuberculata* induced Leaf Anomalies in *R. ferrugineum*.

Leaves of *R. ferrugineum* exhibiting symptomatic infections were systematically gathered during our recent expedition to Shimla, Himachal Pradesh (refer to Figure 2 for visual representation). To preserve the physiological and pathological state of these specimens, each was immediately kept in specialized hermetic bio-storage bags and refrigerated at a precise 4°C.

#### 2.1.2 Sample preparation and segmentation

Under sterile laminar flow conditions, targeted lesions indicative of pathogenic activity was delineated. Using sterilized surgical-grade instruments, these marked sections were excised into consistent, defined segments onto new sterile plates.

#### 2.1.3 Comprehensive surface sterilization

A regimented decontamination process was instituted to obviate exogenous microbial presence. Commencing with an initial rinse in autoclaved distilled water, segments were then briefly exposed to a 75% ethanol solution. Subsequently, a 0.1% (w/v) mercuric chloride (HgCl_2_) immersion ensured a deeper level of sterilization for precisely 1 min. This rigorous sterilization process culminated in a series of three exhaustive washes using sterile distilled water, after which any residual moisture was assiduously removed using autoclaved blotting materials.

#### 2.1.4 Strategic inoculation procedure

Sterilized leaf fragments were gently placed onto specialized Potato Dextrose Agar (PDA) culture plates. To negate bacterial proliferation and maintain a fungus-specific culture environment, Streptomycin sulfate (HIMEDIA’s premium batch TC035-5G) was introduced at an optimized concentration of 100 ppm. This addition takes place before the agar solidifies after sterilization, since the antibiotic becomes inactive at temperatures higher than 55°C.

#### 2.1.5 Monitored incubation

In our state-of-the-art incubation chamber, calibrated to a stable 30°C, the inoculated plates were maintained, fostering a conducive milieu for microbial propagation over an uninterrupted span of 48 h.

#### 2.1.6 Sub-culturing for enhanced growth analysis

Following the initial incubation phase, prominent fungal colonies were selected for sub-culturing. This step involved the transfer of viable growth sections onto fresh PDA plates, each with the aforementioned Streptomycin concentration. The ensuing incubation was monitored and maintained at 30°C until optima fungal sporulation was observed.

#### 2.1.7 Preservation for subsequent analyses

Optimal sporulation was determined through observation and quantification of matured cultures under controlled laboratory conditions. Specifically, sporulation was deemed optimal when a substantial number of mature, well-formed spores were visibly present within the culture. This evaluation included microscopic examination, scrutinizing the abundance, maturity, and uniformity of spores. Additionally, quantitative methods, such as spore counting using a hemocytometer, were employed to ensure a statistically significant number of spores per unit area, with consideration given to a range of 10^5^ to 10^6^ spores per ml. These cultures were carefully stored at 4°C, primed for in-depth morphological, genetic, and pathogenicity studies.

### 2.2 Morphological assessment of the fungal isolate

#### 2.2.1 Sample preparation and transfer

Under strict aseptic conditions, the fungal sample was transferred to a fresh Potato Dextrose Agar (PDA) plate using sterilized instruments. This process guarantees a contamination-free setting, preserving the natural morphological characteristics of the fungi.

#### 2.2.2 Standardized incubation conditions

The newly inoculated PDA plates were carefully positioned inside a Biochemical Oxygen Demand (B.O.D) incubator, a standard for promoting fungal proliferation. The incubation continued for 7 days at a consistent temperature of 30°C. The timeframe was determined on the basis of empirical evidence, ensuring it’s the best duration for the study of this fungal strain’s morphological features.

#### 2.2.3 Detailed colony morphology examination

Upon completion of the incubation, a thorough assessment of the fungal colony ensued. Observations were categorized based on expansion patterns, hue variations, margin intricacies, and notable surface topographies, which could be critical for taxonomic identification.

#### 2.2.4 Microscopic morphological scrutiny

Using a Dewinter compound light microscope, we examined the shape and morphology of the conidial structures. This study focused on attributes like septation, form, size variations, and the identification of any potential vegetative structures like germ tubes and hyphae.

#### 2.2.5 Rigorous spore dimensioning

To ensure statistical relevance, a broad spectrum of approximately 100 spores was methodically selected and assessed. By calculating the dimensions across this array, a reliable average spore size was ascertained, offering a robust metric for morphological comparison.

#### 2.2.6 High-definition photographic cataloging

To retain a permanent record and for future references, high-fidelity microscopic images of the fungi were procured. Scale bars were represented for the microscopic images taken.

#### 2.2.7 Data synthesis and documentation

Carefully collected observations and recorded images were methodically stored. The purpose is to establish a basis for comparing fungi and understanding the isolate’s potential phylogenetic placement.

### 2.3 Pathogenicity test

In our pathogenicity testing aimed at assessing the potential harm to *R. ferrugineum*, a well-structured protocol was deployed. Pristine leaves of *R. ferrugineum* were chosen and placed onto sterilized Petri dishes under an aseptic environment to ensure the purity of the test. Gentle extraction of conidia from the mature fungal colony was done and suspended in autoclaved distilled water until a concentration of precisely 4.6 × 10^6^ spores/ml was achieved. For inoculation, each *R. ferrugineum* leaf was treated with an exact volume of 100 μL of this conidial suspension, ensuring uniform exposure. One leaf that was intentionally left uninoculated served as the negative control, providing a basis for comparison. Following inoculation, the treated leaves were incubated under specific humidity-controlled conditions to create an optimal environment for any potential fungal proliferation. Post the incubation phase, these leaves underwent an intricate microscopic evaluation, employing the high-resolution Dewinter Excel Compound Light Microscope. This was essential to discern and capture the pathogenic interactions at the cellular scale. In accordance with scientific standards, we then re-isolate the fungus from prominent infection spots on the inoculated leaves. This was done in order to fulfill and validate Koch’s postulates, an essential criterion in confirming the pathogenic nature of the organism. Concluding this procedure, the newly isolated fungal strain was juxtaposed with the initial field-obtained specimen. This exhaustive comparison gave insightful details about the plant-pathogen interaction and the threat they might pose.

### 2.4 Detailed genetic profiling through 18S-rRNA and its sequencing protocols

To comprehensively delineate the genetic lineage of the fungal isolate, we executed a meticulous genetic profiling strategy, employing well-established 18S-rRNA and ITS sequencing protocols. Commencing with the isolation of genomic DNA from the cultured fungal specimen, we subjected it to electrophoresis on a 1.0% agarose gel matrix to confirm its purity and integrity. The visualization of a high-molecular-weight DNA band post-electrophoresis attested to the intact genomic DNA without degradation, reflecting the precision of our methodological approach. With confirmed DNA integrity, we progressed to the amplification stage, targeting the Internal Transcribed Spacer (ITS) region—an unequivocal genetic marker in fungal taxonomy. Successful amplification of this region was evident through a distinct amplicon band of approximately 600 base pairs (bp) on an agarose gel post-electrophoresis, employing a polymerase chain reaction (PCR) approach. To ensure the accuracy of our sequencing endeavors, purification of the PCR amplicon was paramount, eliminating residual primers, nucleotides, and potential contaminants. The pristine PCR product underwent bidirectional sequencing using ITS1 and ITS4 primers, executed with precision through the BDT v3.1 Cycle sequencing kit and ABI 3730xl Genetic Analyzer. Subsequent to sequencing, we navigated the realm of bioinformatics, aligning forward and reverse sequences into a refined consensus sequence through sophisticated aligner software. The consensus sequence underwent a BLAST search against the NCBI GenBank database, selecting the top 10 sequences with the highest similarity to our isolate’s sequence.

**Figure 3:**
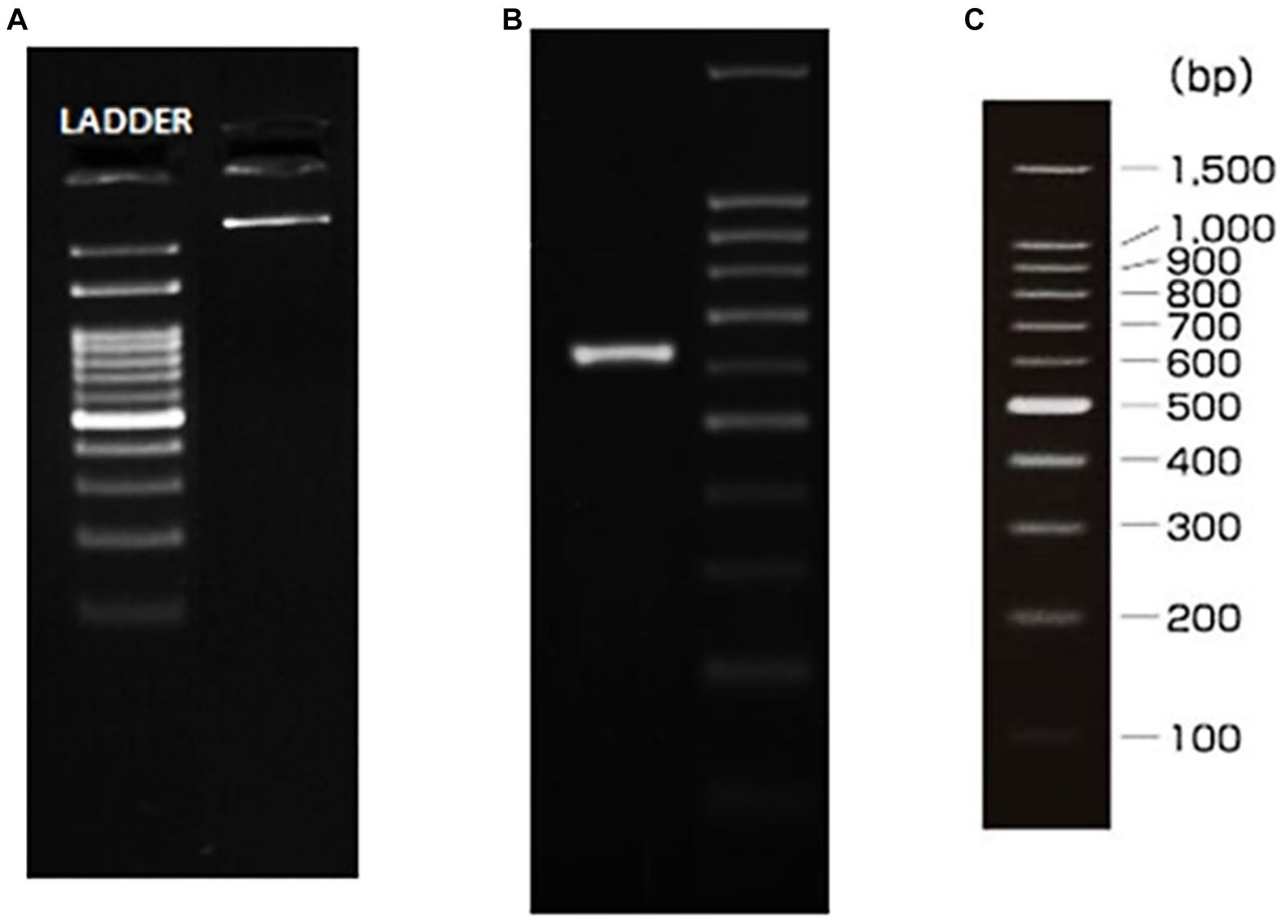
gDNA and ITS amplicon. **(A)** gDNA, **(B)** ITS PCR amplicon, **(C)** Ladder specification.

To unravel the genetic relationship between our isolate and other fungal entities, we utilized ClustalW for multiple sequence alignment, creating a robust alignment of the selected sequences. This alignment served as the foundation for our subsequent phylogenetic analysis. Leveraging the capabilities of MEGA 10 software, we meticulously constructed a distance matrix and a phylogenetic tree, providing profound insights into the evolutionary lineage of our fungal isolate, as depicted in Figure 3 (Wang et al., 2014; Cheng et al., 2016).

### 2.5 Comprehensive phylogenetic examination and bioinformatic interpretation

In our study, the foundational data comprised the ITS sequences of 11 distinguishable *Curvularia* sp., including the newly characterized sequence of *C. tuberculata* OR262505.1 (elaborated in Table 1). Additionally, for phylogenetic interpretation, we utilized 18S rRNA genes. The ClustalW tool, was employed for this intricate genetic juxtaposition (Kumar et al., 2018). This methodical alignment ensures that our subsequent analytical procedures are rooted in precision, facilitating valid interpretations. Post alignment, we subjected the genetic data to an exhaustive evolutionary scrutiny using MEGA11 (Kumar et al., 2018).

**Table 1:** Specifications of the isolates included in the phylogenetic study.

Species	GenBank accession	Isolate	Source	Geographical region
*Curvularia tuberculata*	LC494371.1	12,005	*Oryza sativa*	Taiwan: Tainan City, Liujia Dist.
*Curvularia tuberculata*	MF448225.1	FMB 0005	*Archontophoenix alexandrae*	Pakistan
*Curvularia* sp.	JN207337.1	P41E3	*Cyperus laevigatus*	Venezuela
*Curvularia tuberculata*	ON329689.1	e26	*Oryza sativa*	Mexico: Campeche
*Curvularia tuberculata*	HF934907.1	CBS 146.63	Collected genotyped isolates	Nebraska: Lincoln
*Curvularia tuberculata*	NR_138222.1	CBS 146.63	Collected genotyped isolates	Nebraska: Lincoln
*Curvularia tuberculata*	MN540245.1	L-3106/2012	Human corneal scraping	India
*Curvularia tuberculata*	MH665453.1	DWER3	*Datura wrightii*	India
*Curvularia tuberculata*	OR262505.1	RFP1	*Rhododendron ferrugineum*	India: Himachal Pradesh
*Curvularia tuberculata*	KJ767096.1	A1S2-D16	Beach soil	Malaysia
*Curvularia* sp.	JN207332.1	P36E3	*Eleocharis atropurpurea*	Venezuela

In the subsequent critical phase of our study, we prioritized the Bayesian inference (BI) methodology. Using the PhyML+SMS software suite, BI generated a phylogenetic tree that not only depicted evolutionary trajectories but also maintained a foundation in statistical confidence (Lefort et al., 2017). The application of bootstrapping techniques provided our results with robust statistical support, ensuring that observed phylogenetic relationships among sequences were not coincidental but backed by empirical data. To present our findings in a visually accessible format, we utilized iTOL, a web-based application proficient in crafting interactive phylogenetic depictions (Trifinopoulos et al., 2016). This tool converted our raw data into an easy-to-understand phylogenetic tree, formatted in the widely accepted Newick format. Through this visual representation, we could clearly illustrate the evolutionary distances and complex genetic relationships among the studied *Curvularia* sp. isolates, offering stakeholders a comprehensive and detailed perspective on their genetic heritage (Dhara et al., 2020).

### 2.6 Cold stress-induced sporulation investigation

Initiated from a mature, 7-day old culture, the fungal specimen was meticulously translocated to dual PDA Petri plates, ensuring no external contamination. To simulate cold stress, these plates were methodically sequestered in a cold chamber, maintaining a consistent temperature of 4°C for a span of 8 days. Following this exposure period, a rigorous examination was undertaken. The fungal colonies were treated with Lactophenol cotton blue, a staining reagent renowned for its specificity to fungal structures. This staining regimen rendered the fungal morphology distinctly visible under the high-resolution optics of the Dewinter compound light microscope. To chronicle these observations and capture the nuanced details of sporulation, high-definition micrographs were obtained using a state-of-the-art Dewinter microscope digital camera, DIGI510, which boasts of a 5.1 MP 1/2.5CMOS sensor.

### 2.7 Culture media dependency on fungal growth dynamics

Recognizing the potential influence of culture substrates on fungal growth characteristics, an empirical study was designed to compare the performance of four distinct media formulations. The media chosen for this study encompassed: PDA (HIMEDIA M096, optimally suspended in distilled water at a concentration of 39 g/L), Malt Extract Agar (MEA) (HIMEDIA M1913, with a concentration of 61 g/L), Czapek Dox Agar (CZA) (HIMEDIA M075, meticulously formulated at 49.01 g/L), and Oat Meal Agar (OMA) (HIMEDIA M39, suspended at 72.5 g/L). To ensure uniformity in inoculation and negate potential variability, circular discs with a precise diameter of 6 mm were excised from a pristine, 7-day-old fungal culture utilizing 6 mm stainless steel sterilized cork borers. The discs for inoculation were placed at the center of sterile Petri plates containing the respective growth media. This setup was then incubated at a constant temperature of 30°C. Observations were recorded throughout the incubation period to monitor growth metrics and sporulation rate. Post incubation, for a detailed examination of sporulation patterns, samples were stained and observed under the Dewinter compound light microscope at a magnification of 400X.

## 3 Results & analysis

### 3.1 Comprehensive microscopic characterization of the isolated pathogen

Standard staining techniques and sophisticated, state-of-the-art microscopy tools were used to study the phenotypic and microscopic features of the fungal pathogen isolated from *R. ferrugineum* to determine their micro-morphological features. The deployment of Lactophenol cotton blue staining, renowned for its adeptness in accentuating the nuances of fungal configurations, in tandem with the high-definition capabilities of the Dewinter microscope digital camera (DIGI510, equipped with a superior 5.1 MP 1/2.5” CMOS sensor), yielded intricate insights into the pathogen’s cellular architecture.

**Figure 4:**
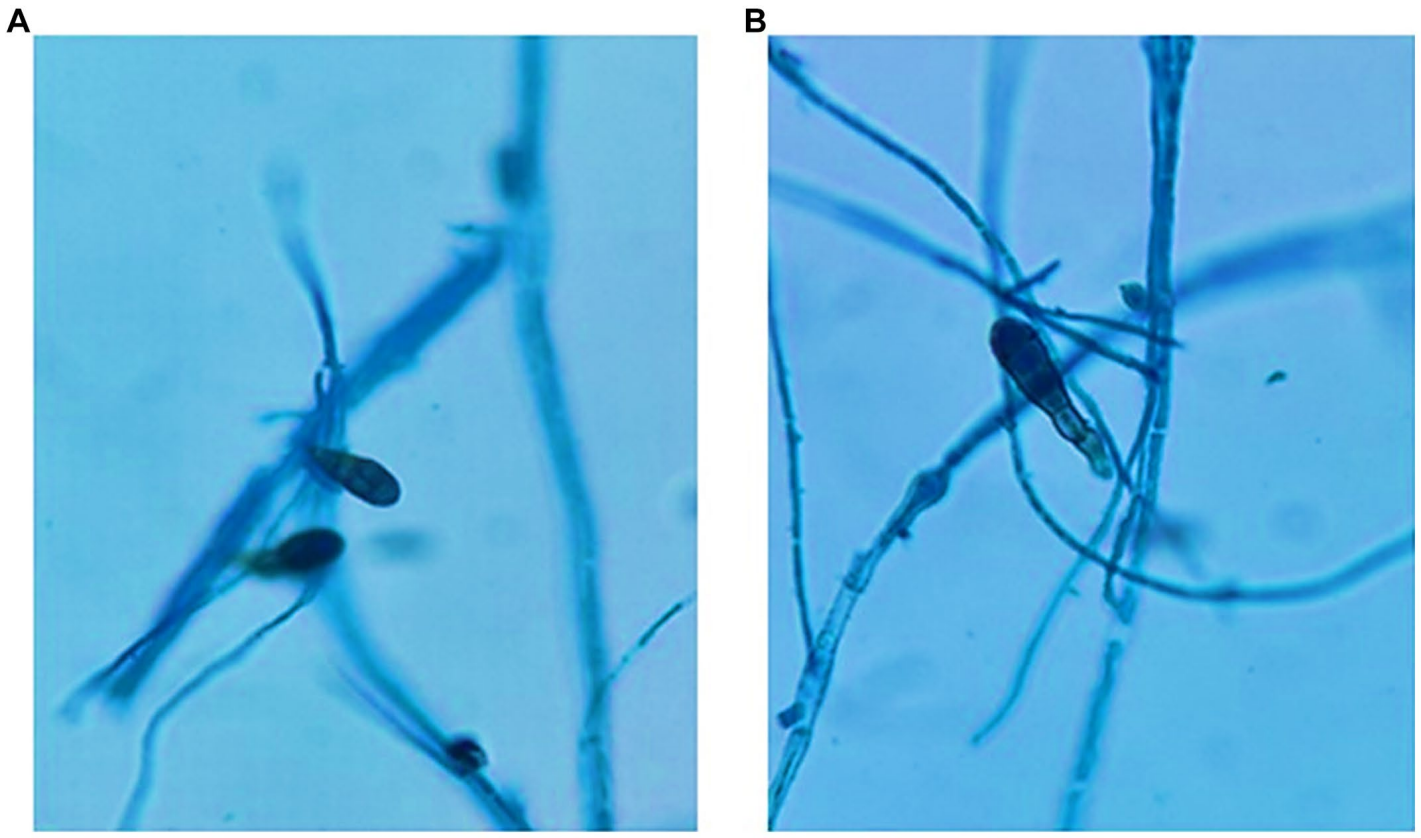
**(A)** and **(B)** Conidial structure of *C. tuberculata* after 7 days of growth in PDA, the fungal hyphae stained with Lactophenol cotton blue stain.

a) Conidial Structural Analysis: Microscopic observations revealed that the conidia exhibited a distinct distoseptate nature. Distinct septation patterns hint towards highly organized cellular mechanism/ s, which are an observable deviation from the conventional fungal septation patterns as shown in Figure 4.
b) Maturation and Septal Distinctions: During the maturation trajectory of the conidia, a notable morphological transformation was evident. Each septum bore darkened transverse bands, rendering a segmented appearance. The central cellular unit showcased heightened pigmentation compared to the terminal cells, potentially alluding to a concentration of vital organelles or compounds. Additionally, this central cell demonstrated a discernible dimensional advantage, being marginally voluminous.
c) Hyphal Dynamics: The fungal hyphae, essential components of the pathogenic structure, exhibited a subtle myelinization pattern. This myelinization, often linked to resilience and environmental adaptability, requires further biochemical exploration. An interesting observation was the distinct conidial attachment method, where a single conidium primarily adhered to the hyphae, indicating a strategic reproductive approach. Additionally, the stalk anchoring the conidium showed robustness, exceeding the thickness of the conidium’s base. By examining these microscopic details, this analysis enhances our understanding of the pathogen’s morphology and highlights the precision offered by contemporary microscopy and staining methodologies. Visual representations supporting these findings are available in Figure 4.

### 3.2 Multifaceted pathogenicity examination

The *in vitro* pathogenicity experiment was executed under rigorous conditions, revealing a panorama of intricate morphological changes on the leaves. Unraveling the landscape of the infection, the leaves exhibited patterns of lesions which ranged from irregular configurations to distinct ellipsoidal shapes. The lesions’ borders emerged with a deep brown hue, encapsulating a gradient transition from buff to a grayish-brown on the lesion’s surface. This particular manifestation potentially mirrors the systematic and aggressive colonization by the pathogen at various developmental stages. In stark contrast, control leaves, untouched by the fungal pathogen, retained their unblemished integrity even after incubation. The experiment involving the application of sterilized water served as a crucial control, confirming its innocuous nature by preserving the leaf’s original morphology. Further microscopic scrutiny revealed more about the pathogen’s nature. The ellipsoidal conidia, which is often an indicator of aggressive fungal strains, was accompanied by a subtle hilum, underscoring the unique morphological attributes of this pathogen.

### 3.3 In-depth insights from ITS sequencing

Embarking on the intricate journey into the pathogen’s genetic blueprint, the ITS sequencing revealed a treasure trove of genomic revelations. Harnessing the power of the BLAST analysis, a striking genomic identity emerged. A near-perfect 99.82% similarity with numerous *C. tuberculata* strains and isolates underscores the taxonomic proximity and genetic fidelity of our isolated pathogen to known strains. Such a high degree of genomic alignment not only bolsters the identification but also accentuates the genetic consistency within this lineage.

### 3.4 Thorough phylogenetic dissection

**Figure 5:**
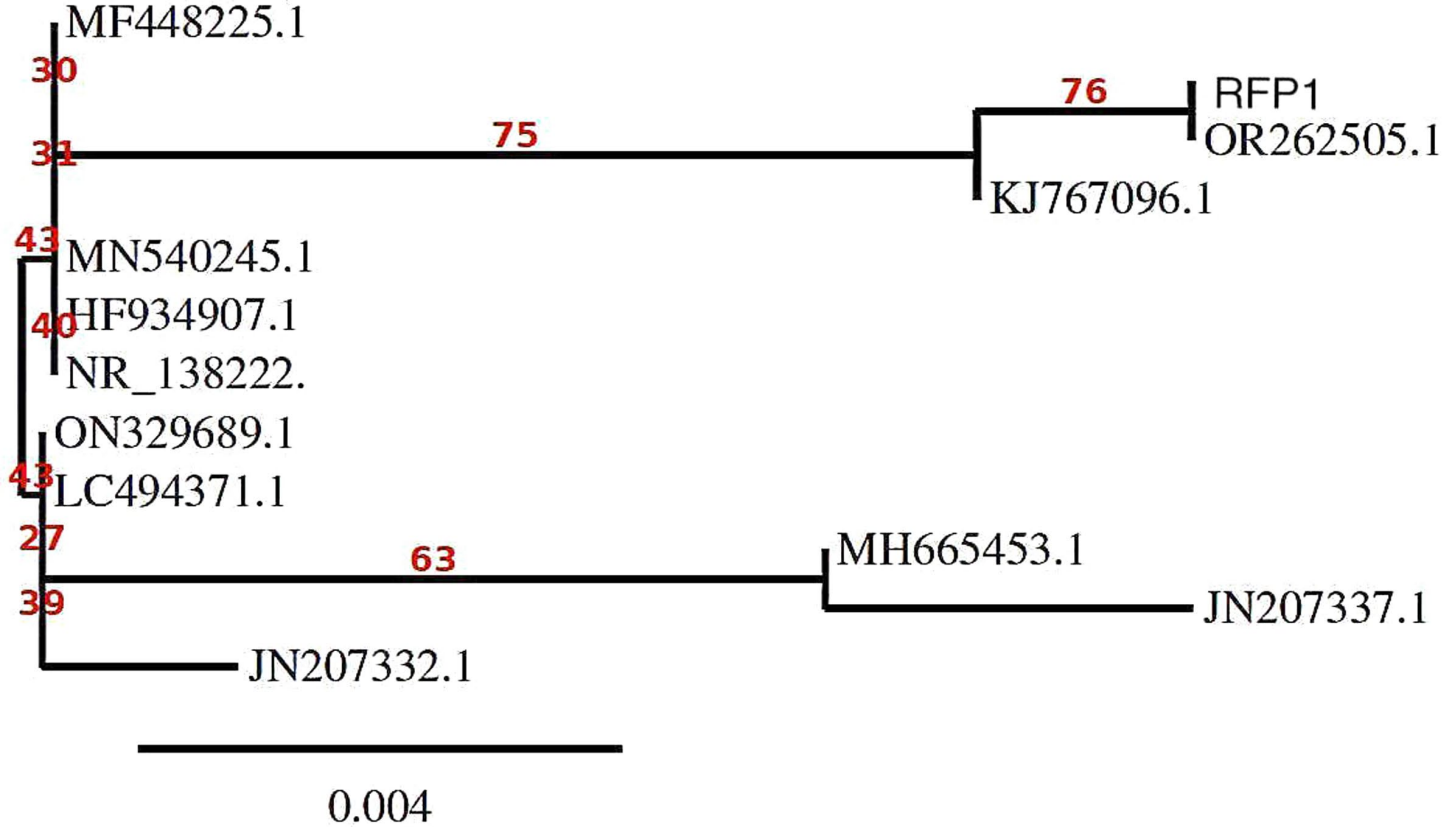
Molecular Phylogenetic analysis by Maximum Likelihood method using MEGA11 software.

Probing the evolutionary tapestry of the strains and isolates through an intensive phylogenetic analysis illuminated diverse genetic pathways and ancestral connections. Our analysis identified two sequences, OR262505.1 and RFP1, as the most closely related within the dataset. Although no bootstrap values were provided for this relationship, their proximity suggests a high degree of relatedness. A significant subtree within the phylogeny, rooted at MF448225.1 with a robust bootstrap value of 80, includes several Curvularia isolates. This subtree encompasses sequences JN207332.1 (bootstrap value: 48), which is closely related to JN207337.1 (bootstrap value: 58) and MH665453.1 (bootstrap value: 34), suggesting a moderately supported cluster. Another branch within this subtree contains ON329689.1 (bootstrap value: 31), which shares a close genetic relationship with LC494371.1 (bootstrap value: 42) and MN540245.1 (bootstrap value: 49), indicating a moderately supported group. NR_138222.1 and HF934907.1 are also closely related, displaying a bootstrap value of 76. KJ767096.1 is positioned as an outgroup, but no bootstrap support was provided, indicating that it is not closely related to the other sequences in the tree (Kimura, 1980). Figure 5 masterfully encapsulates these intricate genetic connections, weaving together a story of divergence, convergence, and co-evolution.

Collectively, this comprehensive exploration offers a holistic perspective on the pathogen’s morphology, genetic identity, and evolutionary trajectory, presenting a detailed narrative that could be pivotal for future studies and interventions.

### 3.5 Cold-induced sporulation dynamics in fungi: an analytical exploration

To delineate the sporulation behavior of the fungal pathogen under a cold-stressed environment, a strategic experiment was instituted, ensuring a controlled yet replicable environment that mirrors potential natural climatic adversities (Wang et al., 2008).

**Figure 6:**
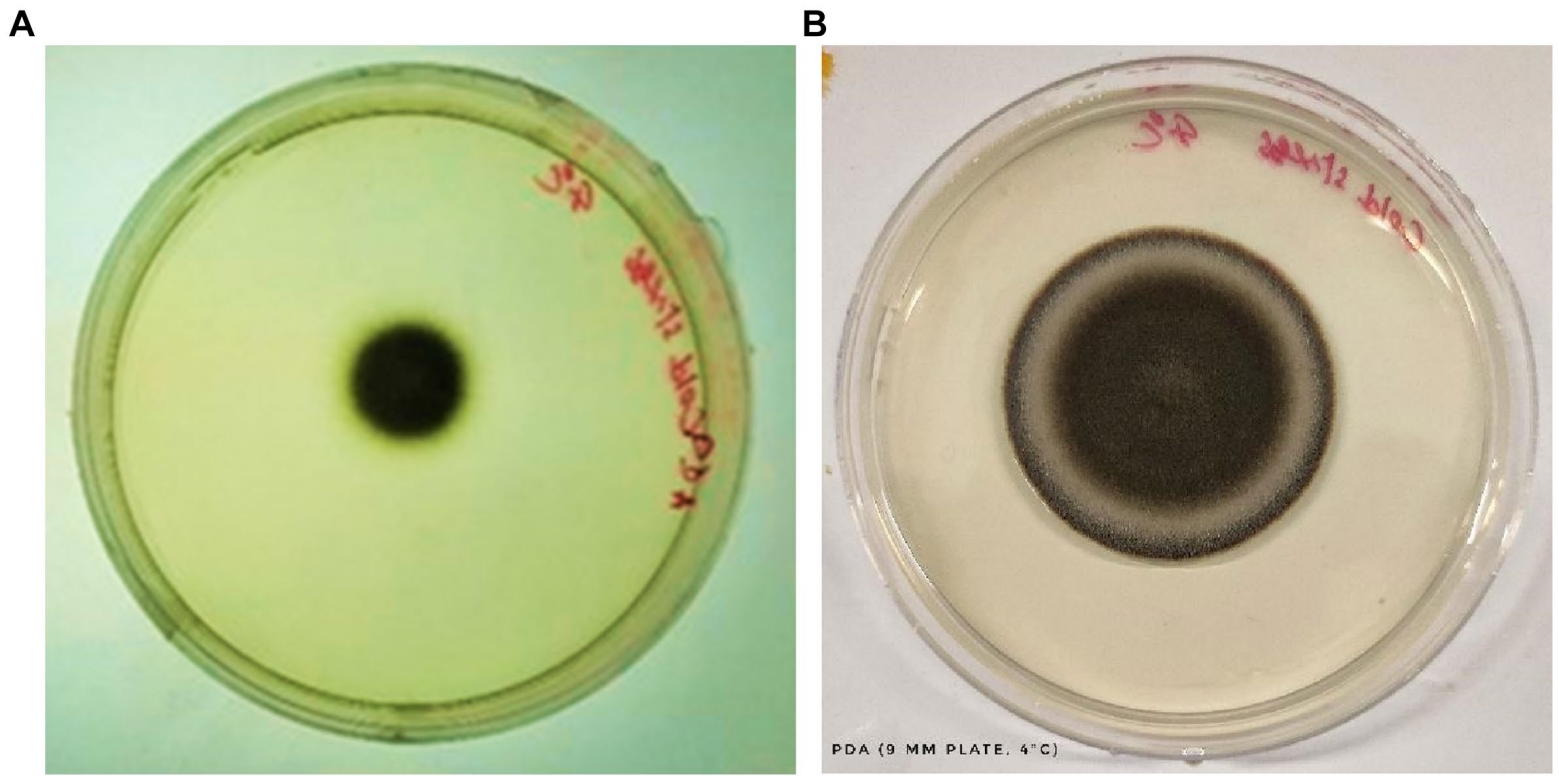
Growth of *C. tuberculata* under cold stress (4°C) after 6 days **(A)** and 8 days **(B)** of incubation, respectively.

a) Colony Dynamics under Cold Stress: Upon 6 days of methodical cold exposure, the ensuing colony dynamics exhibited a marked retardation in overall growth on the Potato Dextrose Agar (PDA) medium. This conspicuous deceleration likely signifies an orchestrated metabolic adjustment, a paradigm that several fungi adopt to economize energy expenditure and channel resources efficiently under non-ideal conditions.
b) Sporulation Proficiency Amidst Adversity: Despite the sub-optimal growth conditions, the fungal pathogen did not compromise its sporulation potential. The discernible presence of a substantive spore population underscores the pathogen’s intrinsic reproductive resilience. The ability to prioritize sporulation even under adverse conditions is indicative of an evolutionary strategy to ensure species perpetuation.
c) Unperturbed Spore Morphology: In a significant observation, the spores, despite being subjected to cold stress, retained their morphological integrity. The cold environment, known to potentially disrupt cellular structures and fluidity in many organisms, appeared to have a minimal perturbative effect on these spores. Such robustness points towards a highly evolved cellular architecture, potentially fortified by protective bio-molecules or mechanisms that confer stability against environmental fluctuations. This morphological conservation is well-documented in Figure 6.
d) Interpretative Synthesis: The cold-induced sporulation dynamics suggests that while growth kinetics might be compromised, the fundamental reproductive modus operandi remains unscathed. The fungus appears to prioritize its long-term survival by investing in sporulation, even under stress. Such adaptive strategies are emblematic of organisms that have undergone evolutionary refinements over millennia, priming them for survival in diverse ecological niches.

The findings from this analysis illuminate a fascinating aspect of fungal adaptability and lay the groundwork for probing deeper into its cellular and molecular defense mechanisms. A more granulated study, possibly employing proteomic or transcriptomic analyses, could unravel the specific pathways and molecules at play during such cold stress responses. This, in turn, can have broad implications not just in ecology but also in applied sciences, especially in biotechnology and agriculture.

### 3.6 Impact of culture media on mycelial growth dynamics

The pivotal role of culture media in modulating fungal growth has always been well-acknowledged. Leveraging this understanding, a comprehensive exploration was undertaken to elucidate how varying culture environments might influence the growth and morphological dynamics of our test fungus.

a) Potato Dextrose Agar (PDA): This media emerged as the clear frontrunner, with the test fungi manifesting a prolific growth rate. With a mycelial expansion reaching an impressive 69 mm over a mere 6-day span, PDA’s nutrient-rich composition seemingly offers an optimal environment, synergizing perfectly with the fungi’s metabolic requisites. This robust growth in PDA is suggestive of a conducive carbohydrate and mineral profile that aligns well with the fungi’s physiological demands.
b) Oat Meal Agar (OMA) and Czapek Dox Agar (CZA): Both media exhibited commendable support for fungal proliferation. With growth metrics settling around 39 mm post a 6-day incubation window, it’s evident that the nutrients available in OMA and CZA cater sufficiently to the fungi’s growth algorithm, albeit not as effectively as PDA. While the exact constituents might differ, the growth suggests that the fundamental requirements of the fungi are being met in both media, leading to almost parallel growth statistics.
c) Malt Extract Agar (MEA): MEA, despite being a widely recognized medium for numerous fungi, displayed somewhat limited compatibility with our test fungus. Clocking in a growth of just 37 mm, this metric is a testament to the specific nutritional preferences or possible inhibitory components inherent in MEA for this particular fungus. However, a silver lining was the spontaneous and pronounced sporulation, hinting that MEA, despite not fostering aggressive vegetative growth, still offers conditions favorable for reproductive maturity.
d) Colorimetric Variations: The morphological canvas, especially the mycelial hue, presented significant diversities across the different media. Such variations could be attributed to differential nutrient assimilation or the influence of specific media constituents on fungal pigmentation pathways. These pigmentation differences not only serve as potential markers for media-specific growth conditions but might also provide insights into any associated physiological or metabolic shifts.
c) Sporulation Dynamics in MEA: Interestingly, while MEA trailed in promoting mycelial proliferation, it surged ahead in catalyzing sporulation. This intriguing dichotomy points towards a possible shift in the fungus’s life strategy - prioritizing reproductive propagation over vegetative expansion when faced with certain nutritional constraints or stimuli.

**Figure 7:**
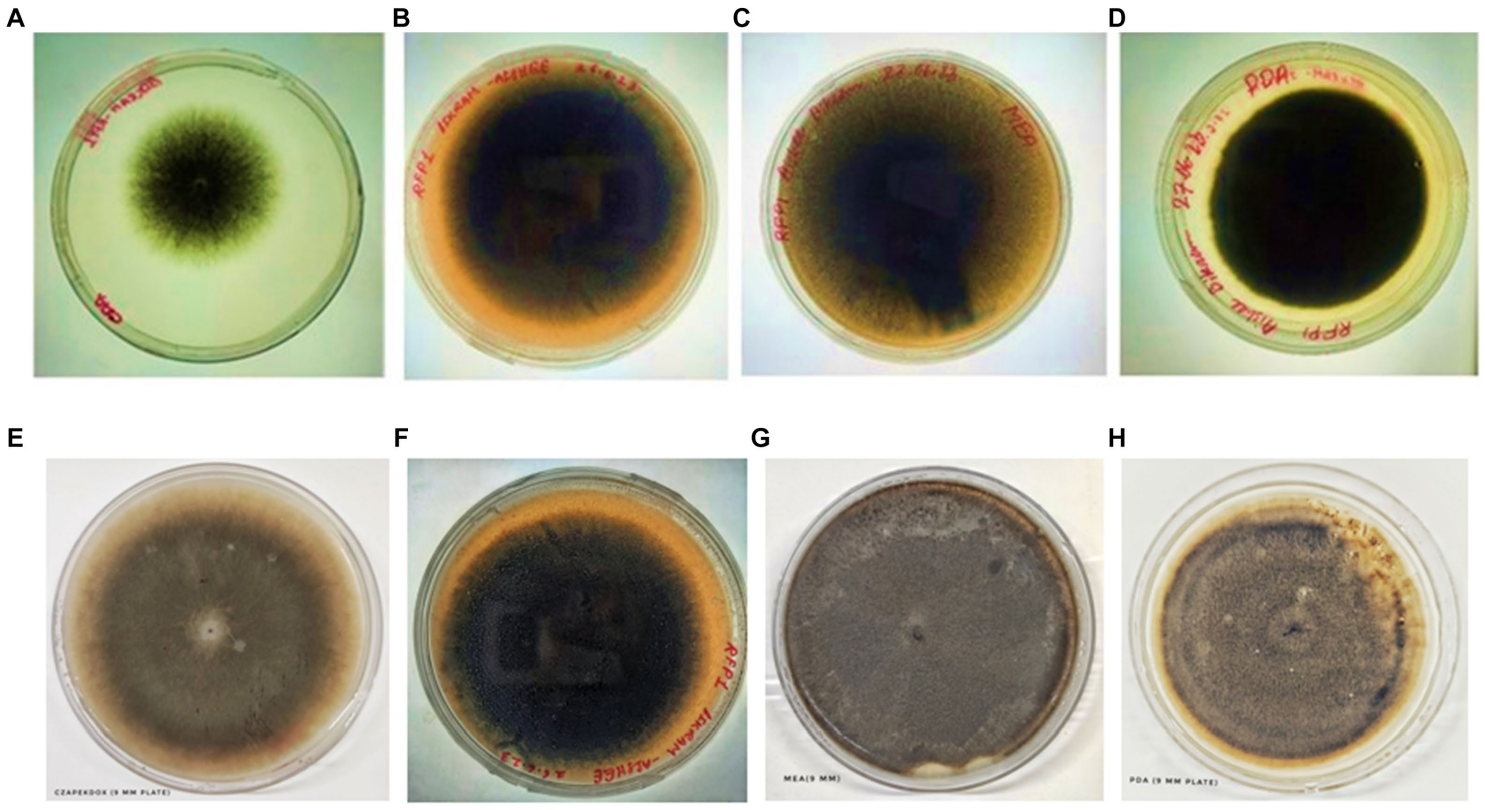
Colony of test fungi in CZA, OMA, MEA, and PDA, respectively, after 4 days of incubation **(A–D)** and 6 days of incubation **(E–H)**.

**Figure 8:**
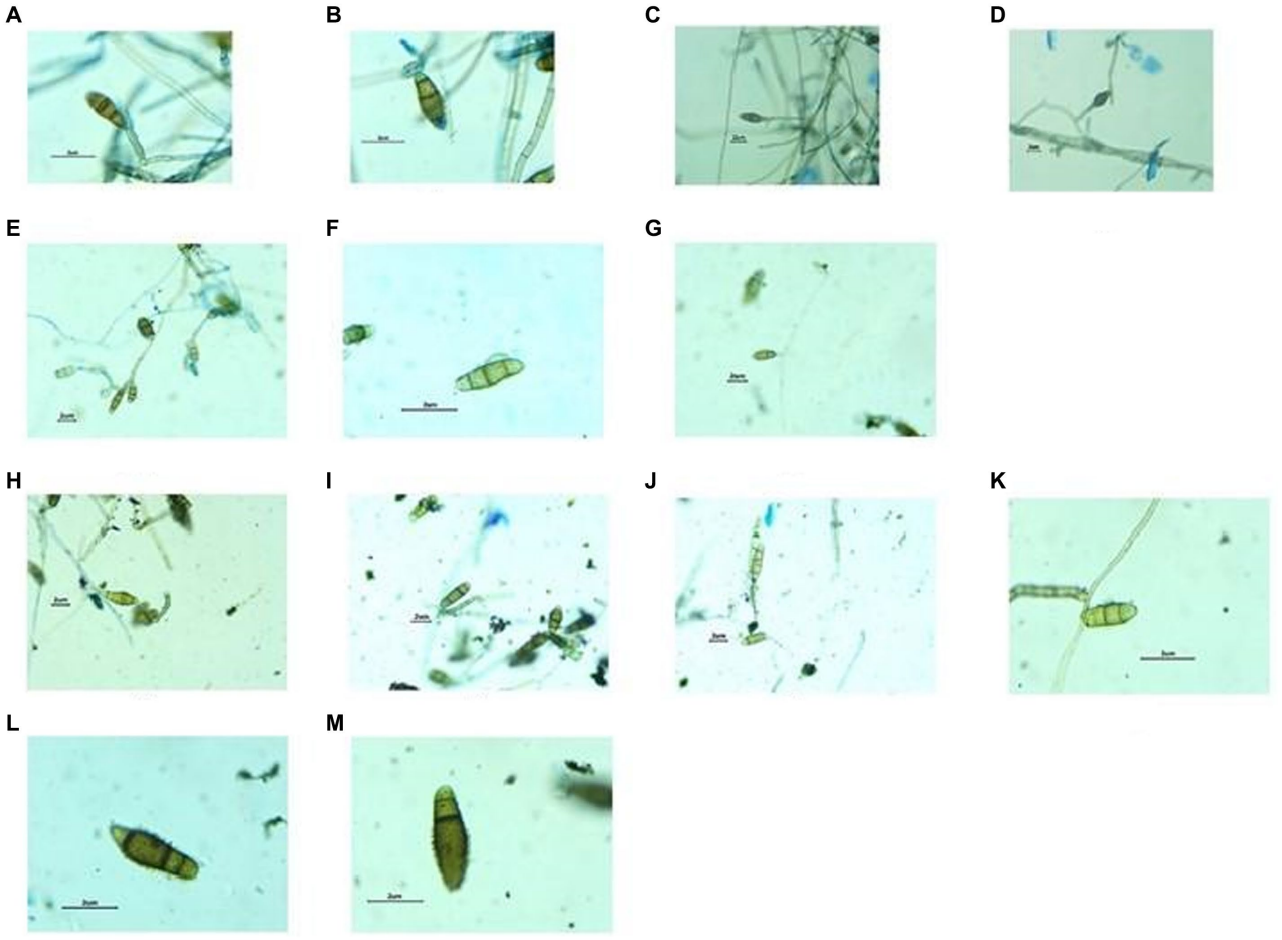
Structures of Fungal hyphae and conidia in different growth media under Dewinter compound light microscope and captured by DIGI510, 5.1 MP1/2.5 “CMOS sensor. Growth in CZA medium, Germinating conidia observed **(A–D)**, Growth in OMA medium, conidia slightly smaller in size **(E–G)** and Growth in MEA medium, Germinating conidia observed **(H–M)**.

The intricate interplay between culture media and fungal growth kinetics has been meticulously examined and vividly illustrated through the extensive analysis conducted in this study. Notably, the choice of culture medium exerts a profound influence on the behavior of the fungal pathogen under investigation, providing insights into its growth patterns and reproductive dynamics. One salient observation from our analysis is the prominence of Potato Dextrose Agar (PDA) as the optimal choice for stimulating sheer vegetative proliferation of the fungal pathogen. PDA, recognized for its nutrient-rich composition, provides an ideal environment for the pathogen’s rapid vegetative growth. This medium’s efficacy in promoting vegetative expansion highlights its utility when the primary research objective is to understand and quantify the pathogen’s ability to proliferate and colonize host tissues. Conversely, our analysis also sheds light on the distinct propensity of Malt Extract Agar (MEA) to stimulate sporulation. MEA, with its unique nutrient composition and specific environmental conditions, serves as a catalyst for the fungal pathogen’s reproductive processes. This medium facilitates the formation of spores, a critical aspect of the pathogen’s life cycle. Researchers keen on exploring the reproductive dynamics, sporulation rates, or aspects related to the pathogen’s life cycle would find MEA to be a valuable choice. In essence, this comprehensive analysis underscores the critical importance of deliberate media selection, contingent upon the specific research objective at hand. Whether the research focus centers on deciphering the nuances of vegetative growth, exploring the intricacies of reproductive dynamics, or encompassing both aspects, as outlined in Table 2 and visually represented in Figures 7, 8, the choice of culture medium becomes a pivotal determinant in shaping the trajectory and outcome of the study. It reinforces the notion that a well-informed decision regarding culture media is paramount in tailoring experiments to address specific research questions and objectives effectively, thus enhancing the depth and breadth of scientific inquiry in the realm of microbial sciences (Table 2; Figures 7, 8).

**Table 2:** Mycelial growth, colony characteristics and sporulation pattern of test fungi on four different culture media.

Media type	Colony diameter (mm)		Colony character		Zonation	Sporulation
	4 days incubation	6 days incubation	Texture	Surface color		
CZA	27.5	39	Furry	Furry black center, basil green colored margin	Concentric	Heavy, conidia germinated
OMA	28	39	Cottony, raised in center	Cottony black colony raised in center, grayish color margin	Concentric	Moderate
MEA	26	37	Cottony	Cottony black colony	Concentric	Heavy, conidia germinated
PDA	58	69	Cottony, raised in center	Cotton black raised in center, creamy white margin	Concentric	Heavy
PDA (Cold Stress)	14	22	Cottony, raised in center	Cotton black raised in center, creamy white margin	Concentric	Heavy

## 4 Discussion

Microorganisms, those diminutive yet immensely influential life forms, occupy an irreplaceable niche within our ecosystems, weaving intricate connections between various facets of human existence and the broader environment. Recent findings, particularly in the domain of pathogenic fungi and their impact on *R. ferrugineum* plants, shed light on the significant implications of these microorganisms on our natural environment. In a thorough and exhaustive study, we have identified a notable issue drawing the attention of horticulturists, botanists, and plant pathologists alike — the emergence of prominent brown blotches on the leaves of *R. ferrugineum* plants. This can be attributed to the presence of the pathogenic fungus *C. tuberculata*, with specific emphasis on the exotype *Curvularia* sp. P41E3. These unsightly blotches, while diminishing the visual appeal of these plants, extend their repercussions beyond aesthetics. They impede the healthy development of flowers and leaves, thereby jeopardizing the ecological and economic significance of *R. ferrugineum* species. This fungal menace, hitherto known to afflict crops like rice and ornamental plants such as the Alexandra palm, has now, for the first time, been documented on *R. ferrugineum* L. plants within the Indian context. Our comprehensive research encompasses a spectrum of methodologies, ranging from the sequencing of the fungal ITS region to cultivating the pathogen in various fungal-specific media. These efforts have yielded deeper insights into the characteristics of this fungus. After aligning the genetic data, we subjected it to thorough evolutionary analysis through MEGA11. This software, revered for its excellence in molecular evolutionary genetics analysis, provides researchers with the capability to explore genetic variances, trace lineages, and discern macroscopic evolutionary patterns inherent in the genetic makeup of the considered *Curvularia* sp. (Kimura, 1980).

In summary, our phylogenetic analysis of *Curvularia* sp. based on ITS sequences reveals distinct relationships among the isolates. OR262505.1 and RFP1 are closely related and likely form a distinct group within the phylogeny. The subtree rooted at MF448225.1 includes sequences with various degrees of relatedness, with moderate support for some relationships, such as JN207332.1, JN207337.1, and MH665453.1. The bootstrap values incorporated into the analysis provide a measure of confidence in the inferred relationships. Higher bootstrap values generally indicate stronger support for the depicted phylogenetic relationships. Therefore, our results suggest that MF448225.1, JN207332.1, JN207337.1, and MH665453.1 are closely related and share a common ancestor, while KJ767096.1 is more distantly related to the other sequences in the tree. These findings contribute to our understanding of the evolutionary history and relationships among *Curvularia* sp., which is valuable for their classification and ecological studies.

Particularly noteworthy is the robust growth exhibited by this pathogen, even in the face of cold temperatures, underscoring its adaptability and the formidable challenges associated with containing its proliferation. This discovery reverberates with the perspective championed at the United Nations General Assembly (UNGA) Science Summit — the imperative of comprehending microorganisms in their entirety. Whether the aim is to harness their beneficial potential or mitigate their adverse effects, the intricate world of microbes stands as a critical frontier of scientific inquiry. The United Nations Sustainable Development Goals (SDGs) resoundingly echo this sentiment, emphasizing the far-reaching influence of microbial sciences on domains as diverse as health, sanitation, agriculture, and more. Microbes, indeed, occupy a pivotal role in numerous sectors. In food production, they facilitate the generation of enzymes, pigments, and flavors, contributing substantially to culinary diversity (Raveendran et al., 2018). In agriculture, they bolster crop yields while concurrently reducing the detrimental impact of chemical inputs, exemplifying their ecological and economic significance. As the global community grapples with escalating health challenges, such as the emergence of new diseases and the paucity of effective drugs, microbes offer a beacon of hope. A multitude of current medications, including anticancer treatments and antibiotics, owe their existence to these microorganisms. The modern era has witnessed their potential as biofactories for innovative drugs and proteins, owing to advancements in recombinant DNA technologies.

Moreover, microorganisms significantly contribute to industrial advancements by producing valuable substances and metabolites. Through omics-led microbial research, we can unlock their potential across diverse fields, from agriculture and health to environmental preservation. In essence, the world of microbes presents both immense opportunities and formidable challenges. While the pathogenic fungi affecting *R. ferrugineum* plants serve as a poignant reminder of the threats posed by microorganisms, the myriad of beneficial applications of these tiny powerhouses signifies their tremendous promise. By investing in a comprehensive understanding of microorganisms and harnessing their potential, we pave the way for a sustainable, prosperous, and harmonious future, where these minuscule entities become indispensable allies in our collective pursuit of global well-being and ecological preservation.

## 5 Conclusion & future perspective

The domain of microbial dynamics, owing to its foundational role within ecological networks, commands profound attention, particularly in light of imperatives surrounding sustainable development. This emphasis has been accentuated by recent deliberations at the United Nations General Assembly (UNGA) Science Summit. Recognizing the intricate interplay between microbial activity and global sustainability challenges, the UNGA Science Summit has ardently championed the intensive study and judicious harnessing of the microbial domain, aligning with the United Nations Sustainable Development Goals (SDGs). Our comprehensive investigation into the adaptive behaviors and growth characteristics of a specific fungal pathogen, within this expansive context, bestows a nuanced dimension upon our comprehension of the practical applications of microbial science. The discernible variations in growth patterns across a diverse array of culture mediums, coupled with the pathogen’s remarkable resilience under stress conditions, serve as a microcosmic representation of microbial robustness and versatility. It is noteworthy that the escalating global emphasis on sustainable agricultural practices and the urgent need to reduce chemical inputs have propelled microbial solutions, such as biofertilizers, into the forefront of agricultural innovation. Our findings offer empirical insights that possess the potential to guide the development of such sustainable microbial interventions with precision and efficacy. Concurrently, within the realm of healthcare, where the world contends with an escalating trajectory of infectious diseases and a diminishing therapeutic arsenal, the phylogenetic nuances unearthed in our study portend promising avenues for targeted drug discovery and development. It bears emphasizing that the pharmaceutical repository is already enriched with microbial derivatives, further underscoring the sector’s potential for groundbreaking innovation.

Beyond these specific domains, the broader implications of our study resonate with global challenges across a diverse spectrum. Whether pertaining to the assurance of water purity through microbial remediation or the harnessing of microbes for alternative energy solutions, our research’s revelations regarding the fungal pathogen’s adaptability symbolize the broader microbial capacity to respond to and potentially mitigate a wide array of ecological and societal challenges. In essence, our study, while delving into the intricate behaviors of a fungal pathogen, also serves as a compelling reminder of the untapped potential within the microbial domain. It provides empirical insights that not only enrich the ongoing scientific discourse but also chart pathways for future interdisciplinary explorations spanning ecology, agriculture, and healthcare. As we find ourselves at the confluence of numerous global challenges, the microbial world, with its vast potential, emerges not merely as an object of curiosity but as an indispensable ally in our unwavering quest for sustainable solutions.

The intricate relationship between microbial dynamics and ecosystem functionality, having only just begun to be unraveled, opens the door to expansive future exploration. The adaptive capacities exhibited by microbes, as exemplified in our study of a specific fungal pathogen, lay the groundwork for designing precise microbial interventions tailored to diverse environmental stressors. These interventions hold the potential to be pivotal in securing sustainable agricultural yields, especially in regions grappling with the rigors of extreme climatic conditions. Furthermore, the pharmaceutical arena holds substantial promise, with potential microbial pathways paving the way for innovative drug development and the formulation of strategies for disease mitigation (Jain et al., 2019). The challenges posed by antibiotic resistance and the increasing incidence of novel diseases underscore the urgency of these prospective endeavors.

Moreover, the burgeoning environmental challenges, spanning from wastewater management to plastic degradation, offer fertile ground for the development of microbe-assisted remediation techniques. The integration of advanced tools, including artificial intelligence and nanotechnology, stands poised to further propel microbial research, advancing us toward solutions that, at present, reside beyond our immediate purview. As we embark upon the new millennium, a resolute focus on microbial sciences promises not only to enrich our scientific repositories but also to equip humanity with innovative tools capable of effectively addressing the multifaceted global challenges that lie ahead. To fully realize this potential, continued dedication to fostering research in this field, coupled with interdisciplinary collaboration, is imperative, ensuring that the microbial world becomes an instrumental partner in our shared endeavor for a sustainable and harmonious future.

## 6 Limitations of the study

While our research has yielded valuable insights into the adaptive behaviors and growth characteristics of the specific fungal pathogen under investigation, it is important to acknowledge certain limitations inherent to our study. These limitations, rather than detracting from the significance of our findings, serve as points of departure for our future research endeavors.

### 6.1 Limited scope of culture conditions

Our study primarily explored the growth patterns of the fungal pathogen across a defined set of culture conditions. These conditions, although representative of key environmental stressors, may not encompass the full spectrum of conditions encountered in nature. Future research will aim to expand the range of culture conditions to provide a more comprehensive understanding of the pathogen’s adaptability.

### 6.2 Limited application scenarios

While our study hints at potential applications in sustainable agriculture and pharmaceutical research, further empirical validation is needed to translate these findings into practical solutions. Future research will focus on rigorous field testing and clinical trials to ascertain the real-world applicability of our discoveries.

### 6.3 Temporal considerations

Our study represents a snapshot in time, and the adaptive behaviors of microorganisms can evolve over time. Conducting long-term studies to track changes in the pathogen’s behavior and its implications on ecosystems is a valuable avenue for future research.

### 6.4 Single-pathogen focus

Our research centered on a specific fungal pathogen, offering valuable insights into its behavior. However, it is essential to recognize that ecological interactions often involve multiple species. Future investigations could delve into the dynamics of microbial communities and their responses to environmental stressors, providing a more holistic view of microbial ecosystems.

### 6.5 Interdisciplinary exploration

The multifaceted nature of microbial dynamics calls for interdisciplinary collaboration. Expanding our research to incorporate insights from diverse fields, such as genetics, ecology, and bioinformatics, will enrich our understanding of microbial behavior and its broader implications.

It is essential to view these limitations as opportunities for further investigation rather than as constraints. They pave the way for our next steps in research, guiding us towards a more comprehensive and nuanced understanding of microbial dynamics and their potential applications. Building upon the foundation laid by this study, our future research endeavors will address these limitations, ultimately contributing to a deeper and more impactful body of knowledge in the realm of microbial science.

## Data availability statement

The datasets presented in this study can be found in online repositories. The names of the repository/repositories and accession number(s) can be found below: NCBI GenBank, OR262502.1-OR262505.1.

## Author contributions

JD: Conceptualization, Formal analysis, Investigation, Methodology, Writing – original draft. AH: Conceptualization, Data curation, Methodology, Resources, Software, Writing – original draft, Writing – review & editing. RP: Conceptualization, Data curation, Investigation, Methodology, Validation, Writing – original draft, Writing – review & editing. VaK: -. VS: Conceptualization, Data curation, Investigation, Methodology, Supervision, Writing – original draft. ViK: Conceptualization, Data curation, Investigation, Writing – original draft. AM: Conceptualization, Formal analysis, Funding acquisition, Investigation, Project administration, Writing – review & editing. AS: Conceptualization, Data curation, Investigation, Methodology, Resources, Writing – original draft. LA: Conceptualization, Data curation, Formal analysis, Methodology, Project administration, Writing – original draft. FE-D: Data curation, Investigation, Project administration, Software, Writing – original draft. MA: Investigation, Software, Validation, Writing – original draft. SA: Conceptualization, Investigation, Validation, Writing – original draft. MA-D: Investigation, Writing – review & editing. AK: Conceptualization, Methodology, Data curation, Software. BD: Conceptualization, Methodology, Software, Formal Analysis, Supervision.
