# Supplementary figures and images for: Unveiling Curvularia tuberculata-induced leaf anomalies in Rhododendron ferrugineum: implications in cultural-ecological conservation and harnessing microbial intervention in socio-economic advancement
Source: Front Microbiol. 2024 Jan 11;14:1280120. doi: 10.3389/fmicb.2023.1280120 (PMC10808759; doi:10.3389/fmicb.2023.1280120)

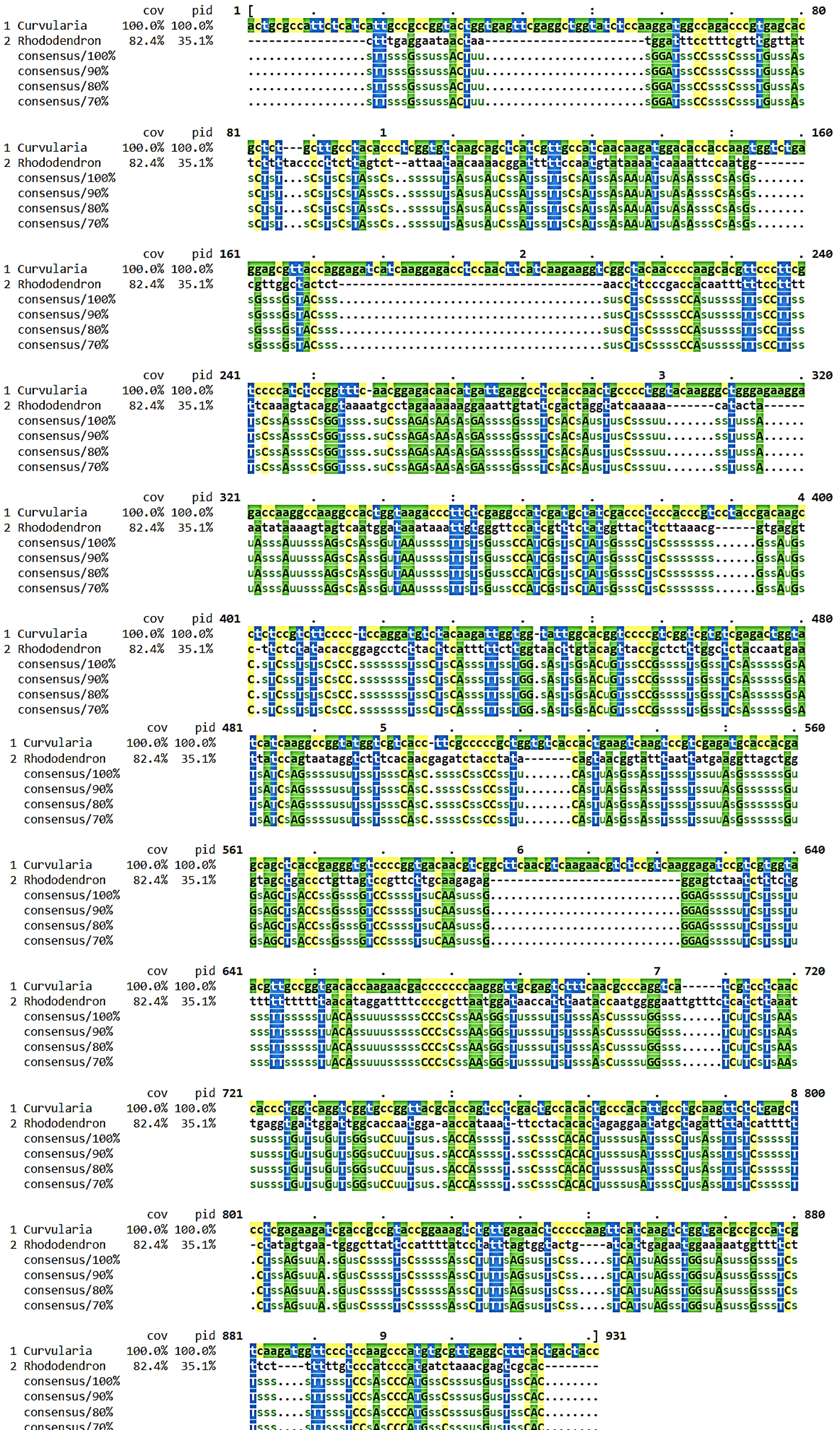

Supplement: Supplementary file 1 [file Image_1.PNG]

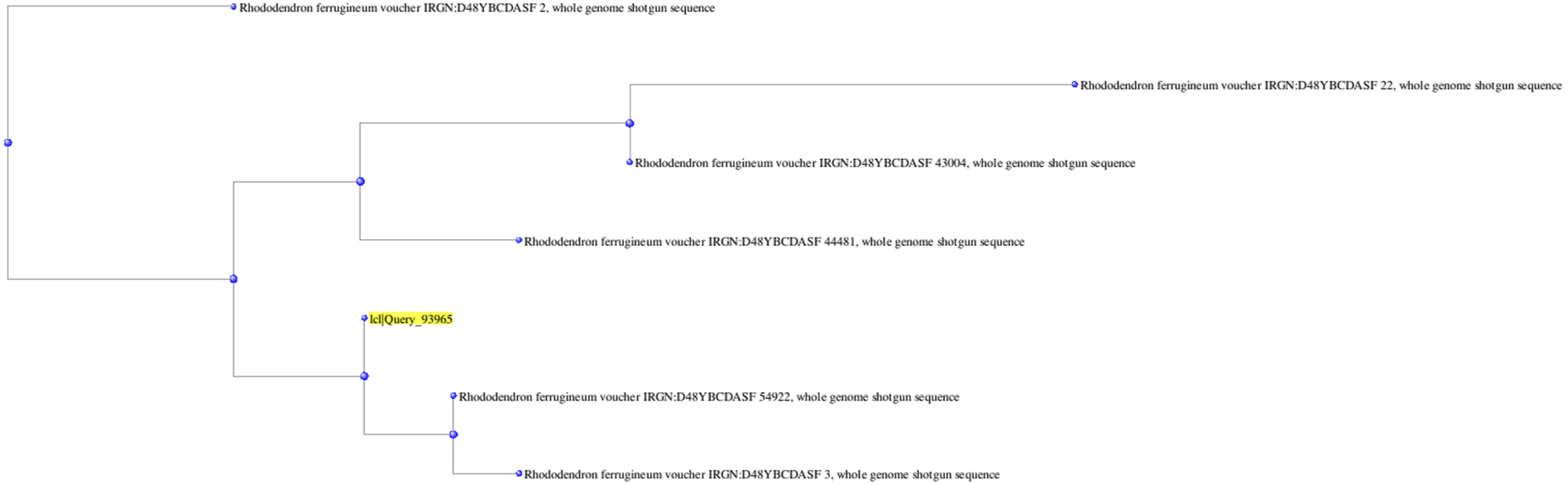

Supplement: Supplementary file 2 [file Image_2.PNG]
